# Active but not inactive granulomatosis with polyangiitis is associated with decreased and phenotypically and functionally altered CD56^dim^ natural killer cells

**DOI:** 10.1186/s13075-016-1098-7

**Published:** 2016-09-13

**Authors:** Wolfgang Merkt, Maren Claus, Norbert Blank, Michael Hundemer, Adelheid Cerwenka, Hanns-Martin Lorenz, Carsten Watzl

**Affiliations:** 1 Department of Hematology, Oncology and Rheumatology, Internal Medicine V, University Hospital of Heidelberg, Heidelberg, Germany; 2 Leibniz Research Center for Working Environment and Human Factors at TU Dortmund (IfADo), Ardeystraße 67, Dortmund, 44139 Germany; 3 Innate Immunity Group, German Cancer Research Center, Heidelberg, Germany

**Keywords:** Granulomatosis with polyangiitis, Natural killer cells, Vasculitis, Fc receptor, CD16, NKG2C, CCR5, CD54, Natural cytotoxicity

## Abstract

**Background:**

The role of natural killer (NK) cells in granulomatosis with polyangiitis (GPA) is poorly understood. We recently reported that peripheral blood NK cell percentages correlate with the suppression of GPA activity (cohort I). The purpose of the current study was to further characterize NK cell subsets, phenotype and function in a second GPA cohort (cohort II).

**Methods:**

Peripheral blood lymphocyte subsets were analyzed at a clinical diagnostic laboratory. Clinical data were extracted from medical records and patients were grouped according to their activity state (remission vs. active/non-remission). Separate analysis (cohort II, *n* = 22) and combined analysis (cohorts I and II, *n* = 34/57) of NK cell counts/percentages was performed. NK cell subsets and phenotypes were analyzed by multicolor flow cytometry. Cytotoxicity assays were performed using ^51^Cr-labeled K562 target cells.

**Results:**

In cohort II, NK cell counts were lower than the lower limit of normal in active GPA, despite normal percentages due to lymphopenia. NK cell counts, but not other lymphocyte counts, were significantly higher in remission. Combined analysis of cohorts I and II confirmed decreased NK cell counts in active GPA and increased percentages in long-term remission. Follow-up measurements of six patients revealed increasing NK cell percentages during successful induction therapy. Multicolor analysis from *cohort II* revealed that in active GPA, the CD56^dim^ subset was responsible for decreased NK cell counts, expressed more frequently CD69, downregulated the Fc-receptor CD16 and upregulated the adhesion molecule CD54, the chemokine receptor CCR5 and the activating receptor NKG2C. In remission, these markers were unaltered or marginally altered. All other receptors investigated (NKp30, NKp44, NKp46, NKG2D, DNAM1, 2B4, CRACC, 41BB) remained unchanged. Natural cytotoxicity was not detectable in most patients with active GPA, but was restored in remission.

**Conclusions:**

NK cell numbers correlate inversely with GPA activity. Reduced CD56^dim^ NK cells in active GPA have an activated phenotype, which intriguingly is associated with profound deficiency in cytotoxicity. These data suggest a function for NK cells in the pathogenesis and/or modulation of inflammation in GPA. NK cell numbers, phenotype (CD16, CD69, NKG2C) or overall natural cytotoxicity are promising candidates to serve as clinical biomarkers to determine GPA activity.

**Electronic supplementary material:**

The online version of this article (doi:10.1186/s13075-016-1098-7) contains supplementary material, which is available to authorized users.

## Background

Natural killer (NK) cells make up about 5–15 % of all peripheral blood lymphocytes (PBL). They are tightly regulated lymphocytes with cytotoxic activity against stressed and/or antibody-coated cells [1–4]. Moreover, NK cells link innate and adaptive immunity [1]. Adaptive immunity can be modulated by NK cells at different stages [5], e.g. by secretion of interleukin (IL)-10 [6] and killing of other immune cells, such as activated CD4+ T-cells [7]. Two major NK cell subsets are well-established: CD56^bright^ NK cells are dominant in secondary lymphatic tissues and presumably precursors of CD56^dim^ NK cells, which are mature cytotoxic cells and make up >90 % of blood NK cells. CD56^dim^ NK cells can be further subdivided based on the expression of CD62L and CD57 [2]. Important homeostatic factors are IL-15 and IL-18 [8].

NK cell immune recognition and activity is balanced by activating and inhibiting receptors [9], but can be modulated on several levels [2]. CD56^dim^ NK cells bear the low affinity Fc-γ-receptor CD16 (FcγRIIIA). CD16 binds IgG1 and mediates antibody-dependent cellular cytotoxicity (ADCC) [10]. CD16 plays a prominent role in activating CD56^dim^ NK cells [11]: CD16 activates resting NK cells by itself, whereas all other receptors need co-activation. Following stimulation, CD16 is downregulated [12] by means of shedding [13] and intracellular uptake [14].

Granulomatosis with polyangiitis (GPA) is a systemic inflammatory, prototype autoimmune disease characterized by the presence of anti-proteinase-3 autoantibodies. Granulomas and vasculitis histologically define GPA. Knowledge about the involvement of NK cells in GPA is limited. We have recently discovered that blood NK cell percentages correlate negatively with disease activity and positively with the duration of remission [15]. NK cells were not detectable in active granulomatous lesions [15]. To date, alterations in the phenotype or function of NK cells have not been linked with disease activity.

## Methods

### Patient consent and ethical approval

Informed consent was obtained from the patients before study initiation. The ethics committee of the University of Heidelberg approved this study.

### Patients and controls

Two GPA patient cohorts were analyzed. Details of cohort I have been published [15]; 28 different patients were included. Blood was taken at different time points from 4/28 patients, so that 35 measurements were performed in total (NK cell percentages in PBL). Cohort II consisted of 19 different patients, in total providing 22 PBL subset measurements and 19 analyses of phenotype. Characteristics of patients in cohort II are specified in Table 1. In both cohorts a total of 57 measurements were analyzed. Multiple measurements of the same patients with GPA were included, as the activity state might have been different (see below).

**Table 1:** Patient characteristics (cohort II and CD*)

Characteristic	Value
Patients with GPA, *n*	22
Men/women, *n*	14/8
GPA in remission, *n* (%)	12/22 (55 %)
Age in years, median (range)	55.5 (35–79)
Duration of remission in years (of inactive GPA), mean (range)	4.4 (1–20)
GPA non remission (active), *n* (%)	10/22 (45 %)
Age in years, median (range)	51.5 (33–64)
BVAS, mean (range)	4.5 (0–19)
Localized GPA (upper airways and ENT organs only), *n* (%)	4/22 (18 %)
Generalized GPA, *n* (%)	18/22 (82 %)
ANCA	
Positive	17/22 (77 %)
Negative	3/22 (14 %)
Not determinable	2/28 (9 %)
Patients with CD, *n*	12
Men/women, *n*	3/9

*GPA* granulomatosis with polyangiitis, *BVAS* Birmingham vasculitis activity score, *ENT* ear, nose and throat, *ANCA* antineutrophil cytoplasmic antibody, CD* inactive systemic inflammatory control diseases other than GPA comprising systemic lupus erythematosus (*n* = 4)*,* panarteriitis nodosa (*n* = 1), overlap connective tissue disease (*n* = 1), chronic inflammatory bowel disease (*n* = 1), CREST syndrome (*n* = 1), primary Sjögren’s syndrome (*n* = 1), polymyalgia rheumatica (*n* = 1) and giant cell arteriitis (*n* = 2)

### Analysis of lymphocyte subsets (cohort II)

**Fig. 1:**
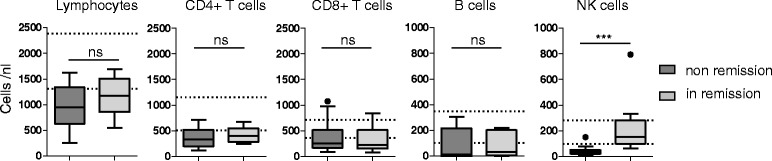
Natural killer (*NK*) cell counts are significantly lower in active (non-remission) granulomatosis with polyangiitis. Peripheral blood lymphocyte counts and lymphocyte subsets were determined by flow cytometry (clinical grade quality), as indicated in the figure. Only patients from cohort II were included (total patients = 22, patients with active disease = 10, patients in remission = 12). Boxplots show traditional Tukey *whiskers*; *dotted lines* show upper and lower limits of normal. Statistical analysis was performed using the Mann-Whitney test; *ns* not significant; ***p = 0.0005

Lymphocyte subsets in 22 samples from 19 different patients were analyzed on the same day of blood donation in a clinical diagnostic laboratory using a standard antibody kit (clinical grade quality) from Beckman Coulter (Brea, CA, USA). We did not perform this test with a healthy control group, as upper and lower limits of normal are already established for clinical diagnostics (see dotted lines in Fig. 1).

### Definitions and analysis of medical records

Medical records from all patients from cohorts I and II were (re-)analyzed. To best reflect clinical activity, the definitions of GPA activity states from current recommendations for clinical studies [16–19] were slightly adapted and applied as follows: remission on drug - inactive disease, absence of symptoms for at least 6 months, prednisone intake <10 mg/day; remission off drug - inactive disease, absence of symptoms, no disease-modifying antirheumatic drug (DMARD) intake for at least 6 months. By definition, the Birmingham vasculitis activity score (BVAS) had to be 0 to identify remission; response was defined as BVAS reduction >50 % compared to the last clinical visit; refractory disease was defined as no response after 6 weeks of treatment or progressive disease after 4 weeks of treatment, or chronically persisting activity with one major or three minor BVAS items; grumbling disease was defined as minor, non-organ-threatening symptoms that usually respond to elevation of prednisone doses or DMARDs (e.g. arthralgia, myalgia or fever); major relapse was defined as one major or three minor BVAS items; minor relapse was defined as recurrent disease activity without organ-threatening manifestations, and early relapse was defined as relapse <6 months after initiation of induction therapy.

The activity state and BVAS were determined at the time of blood donation, i.e. the day of inclusion in the study. To facilitate the analysis, on-drug and off-drug remission were grouped into a single group classified as in remission. All other activity states were termed active or non-remission. Please note that in remission requires the absence of symptoms (i.e. BVAS of 0) for at least 6 months, therefore patients with a BVAS of 0 can principally be allocated to the non-remission group. The definition for short-term and long-term remission (< vs. >/= 4.08 years) was adopted from the parental study [15]. Physician global assessment was categorized as active, inactive or diverse; the diverse category includes uncertainties and ambiguities (e.g. increased use of prednisone by the patient because of unspecific arthralgia). Therapeutic consequence was categorized as intensified, unchanged or reduced, according to the physician’s decision about the use of immunosuppressive pharmaceutical treatment.

### NK cell phenotyping/multicolor flow cytometry

Peripheral blood mononuclear cells (PBMCs) from patients (cohort II; in remission, *n* = 11; active, *n* = 8; total, *n* = 19;) and nine healthy controls (HC) were isolated by Ficoll-Paque density gradient centrifugation using Pancoll medium (PAN-Biotech GmbH, Aidenbach, Germany) and deep-frozen the same day. Samples were thawed and 200,000 PBMCs were incubated for 20 minutes on ice in Zombie aqua (Biolegend, San Diego, CA, USA) dissolved in PBS, washed, incubated for 20–30 minutes on ice with a cocktail of monoclonal antibodies (panels, see Additional file 1: Methods), then washed and directly analyzed on a four-laser flow cytometer (LSR Fortessa, BD Biosciences, San Jose, CA, USA). Data were processed using FlowJo® software (FlowJo LCC, Ashland, OR, USA). The following mean counts of live CD56^dim^CD16pos. NK cells were analyzed (panel 2): 15,000 (HC); 8,000 (GPA in remission) and 3,000 (GPA non-remission). The lowest counts of live analyzed CD56^dim^CD16pos. NK cells were 307 and 446 within the GPA non-remission group; all others were >1,500 per measurement.

### Cytotoxicity assay

The target cell line K562 was grown in medium (IMDM, 10 % FCS, 1 % penicillin/streptomycin) to mid-log phase. 5 × 10^5^ target cells were labeled in 100 μL assay medium (IMDM with 10 % FCS and penicillin/streptomycin) with 100 μCi of ^51^Cr for 1–2 hours at 37 °C. Cells were washed twice in assay medium and resuspended at 5 × 10^4^ cells/mL in assay medium. Five thousand target cells/well were used in the assay. Effector cells (freshly isolated, thawed PBMCs) were resuspended in assay medium and left untreated overnight. PBMC viability was then analyzed using CASY® Cell Counter (OMNI Life Science GmbH & Co. KG, Bremen, Germany). PBMCs were mixed with labeled target cells in a V-bottom 96-well plate. Maximum release was determined by incubation in 1 % Triton X-100. For spontaneous release, target cells were incubated without effector cells in assay medium alone. All samples were analyzed in triplicates. Plates were incubated for 4 hours at 37 °C. Supernatant was harvested, and ^51^Cr release was measured in a gamma counter. Percentage of specific release was calculated as:

((Experimental release – Spontaneous release)/(Maximum release – Spontaneous release)) × 100.

### Statistical analysis

Exploratory statistical analysis was performed; *p* values have to be interpreted descriptively. Normal distribution was not assumed; non-parametric statistical tests were used. The Kruskal-Wallis test and Dunn's post hoc test were used for multiple comparisons; the Mann-Whitney test was used to compare two patient groups; Spearman’s test was used to test for correlation. The Wilcoxon signed rank test was used to compare NK cell proportions from the same donors at different time points. All tests were performed with a significance level of 5 % (confidence interval 95 %).

## Results

### NK cell counts were significantly lower in active (non-remission) GPA

Lymphocyte subsets in 22 samples from 19 different patients in cohort II were analyzed. Patients with GPA had lymphopenia, irrespective of disease activity (Fig. 1). In active GPA, lymphopenia resulted from collectively reduced T, B and NK cells. Numbers of NK cells were markedly low; a median of 33.5 NK cells/nl corresponded to 1/3 of the lower limit of normal. On statistical analysis using the Wilcoxon signed rank test, NK cell counts from non-remission GPA were significantly lower than a hypothetic value of 188.5 (the mean of the lower and upper threshold of normal NK cell counts; *p* = 0.002). Interestingly, in remission, NK, but not T or B cell counts, were in the normal range and were statistically significantly higher than in active GPA.

### NK cells were inversely correlated with GPA activity

**Fig. 2:**
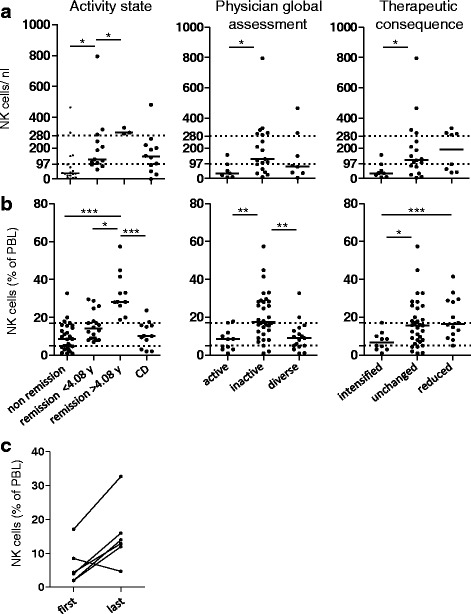
Natural killer (*NK*) cells inversely correlate with granulomatosis with polyangiitis (GPA) activity. Patients from cohort I and II were included; cohort I has been published in a parent study [15]; cohort II is described in Table 1. **a** Absolute NK cell numbers are decreased in active GPA. Cohort I, *n* = 12; cohort II, *n* = 22; total = 34; control disease (*CD*), *n* = 12 patients with inactive systemic inflammatory control diseases (see [15] and Table 1). *Dotted lines* show upper and lower limits of normal NK cell numbers according to our clinical diagnostic laboratory; medians are indicated by bars. *Left* subgrouping according to activity states showed significant differences among the groups (Kruskal-Wallis test, *p* = 0.0029); *middle* physician global assessment (Kruskal-Wallis test, *p* = 0.0486); *right* therapeutic consequence (Kruskal-Wallis test, *p* = 0.0397). **b** NK cell percentages are increased in patients with GPA in long-term remission. Cohort I, *n* = 35; cohort II, *n* = 22; total = 57; CD, *n* = 12. *Dotted lines* correspond to the upper and lower limits of normal NK cell percentages, according to our clinical diagnostic laboratory; medians are indicated by bars. *Left* subgrouping according to activity states showed significant differences among the groups (Kruskal-Wallis test, *p* = 0.0001); *middle* physician global assessment (Kruskal-Wallis test, *p* = 0.0007); *right* therapeutic consequence (Kruskal-Wallis test, *p* = 0.0108). **a**, **b** The threshold for long-term remission (4.08 years) had been determined in the parent study [15]. Dunn's post hoc test was significant where indicated in the graphs. **c** NK cells increase during successful induction therapy. NK cell percentages from six patients with GPA were determined at two or more time points during induction therapy (cohort I, four patients; cohort II, two patients). The first and last measurements are depicted, respectively. Statistical analysis was performed using the Wilcoxon signed rank test, *p* = 0.0585

Both NK cell lymphopenia in active GPA and the significantly lower NK cell counts in active vs. inactive GPA were confirmed in a combined analysis of cohort I (*n* = 12) and cohort II (n = 22; total = 34) after subgrouping according to either BVAS (>/= 0, *p* = 0.0152, not shown) or activity state (Fig. 2a, left graph). Likewise, analysis of physician global assessment and therapeutic consequence showed reduced NK cell counts in active vs. inactive GPA (Fig. 2a, middle and right graphs). The majority of patients with non-remission GPA had NK cell counts lower than the clinically established threshold of "normal". When comparing non-remission GPA with inactive control disease (CD) (Fig. 2a), the corresponding post hoc test was not significant, whereas the direct comparison using the Mann-Whitney test was significant, indicating borderline significance. The latter is presumably due to the relatively low number of patients with CD and the large range of scattered NK counts within the CD group.

In the combined analysis, the total of 57 measurements of blood lymphocyte percentages reproduced the previous finding of increased NK cell percentages during remission [15]. NK cell percentages in long-term remission were higher than usually seen in healthy individuals (Fig. 2b, left graph, and [15]). Likewise, on analysis of physician global assessment and therapeutic consequence there were increased NK cell percentages in inactive vs. active GPA (Fig. 2b, middle and right graphs).

These data indicate that NK cells increase during the transition from active to inactive GPA. We assessed NK cell percentages in six patients at least twice during the course of induction therapy. In 5/6 patients, NK cell percentages increased during successful induction therapy (Fig. 2c). In 1/6 patients, NK cell percentages were lower in the last measurement. This patient had high initial disease activity (BVAS 2006 = 23), suffered from early relapse between the first and the last measurement (BVAS 2006 = 24), and finally responded to therapy with persistent activity at the last measurement (BVAS 2006 = 12). Absolute NK cell counts were determined in 4/6 patients, showing a minor increase in 3/4 patients (not shown, not statistically significant). Furthermore, we had the chance to observe patients who experienced clinical deterioration during follow up; all four patients had decreased NK cell percentages compared to the previous value (not shown, not statistically significant).

These data extend our previous study showing that NK cell numbers are lower than normal in active GPA and normalize during clinical amelioration. The finding that NK cell percentages further increase during long-term remission is confirmed (by additional patients from cohort II and by the new analysis methods). NK cell percentages are likely to reflect disease activity within a given patient.

### CD56^dim^ but not CD56^bright^ NK cells were reduced in active GPA

We established a set of multicolor flow cytometry panels to investigate NK cell subsets and important surface proteins. PBMCs from 18 different patients in cohort II and 9 healthy controls (HC) were isolated by density gradient centrifugation and frozen. Except for one patient, no double measurements were included (total = 19, remission = 11 and active = 8 patients). The age was not significantly different between HC and patients in remission and non-remission (Additional file 2: Figure S3). NK cells were defined as viable CD3-negative CD56-positive lymphocytes.

**Fig. 3:**
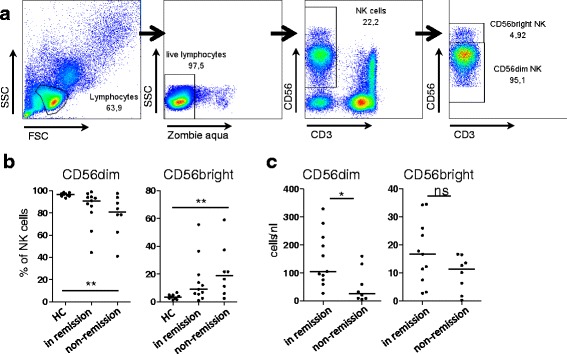
CD56^dim^ but not CD56^bright^ natural killer (*NK*) cells are reduced in active (non-remission) granulomatosis with polyangiitis (GPA). Peripheral blood mononuclear cells from patients with GPA (total = 19, in remission = 11 and non-remission = 8 patients) and 9 healthy controls (*HC*) were isolated by density gradient centrifugation and analyzed by multicolor flow cytometry (panel III). **a** Gating strategy. Peripheral blood lymphocytes were determined using forward scatter (*FSC*) and side scatter (*SSC*). Cell viability was assured by negative Zombie aqua staining. NK cells were determined by co-staining of CD3 (negative) and CD56 (positive). **b** Percentages of CD56^dim^ (left) and CD56^bright^ (*right*) NK cells among total NK cells were plotted according to activity states. The Kruskal-Wallis test was significant in both graphs (*p* = 0.0084 and *p* = 0.0081), Dunn's post hoc test was significant where indicated in the graphs; *bars* represent medians. **c** Absolute numbers of CD56^dim^ (*left*) and CD56^bright^ (*right*) NK cells were calculated on the basis of the absolute NK cell counts shown in Fig. 1. The Mann-Whitney test was significant only for CD56^dim^ NK cells (*p* = 0.0149); *bars* indicate medians. *ns* not significant

We first focused on traditional NK cell subsets. As we had not found differences in the distribution of CD56^dim^ and CD56^bright^ NK cells in the parent study [15], we used another, more sensitive anti-CD56 antibody (Fig. 3a). We found that the percentages of CD56^dim^ NK cells among total NK cells were lower in active GPA, whereas CD56^bright^ NK cell percentages were increased (Fig. 3b). On analysis of absolute numbers the CD56^dim^ NK cells were significantly lower in active GPA than in remission, whereas the counts of CD56^bright^ NK cells were not significantly different (Fig. 3c). Therefore, a decrease in the CD56^dim^ subset was responsible for the decreased total NK cell numbers in active GPA.

### CD56^dim^ NK cells in active GPA express high levels of lymphocyte activation marker CD69 and low levels of Fc-gamma receptor CD16

**Fig. 4:**
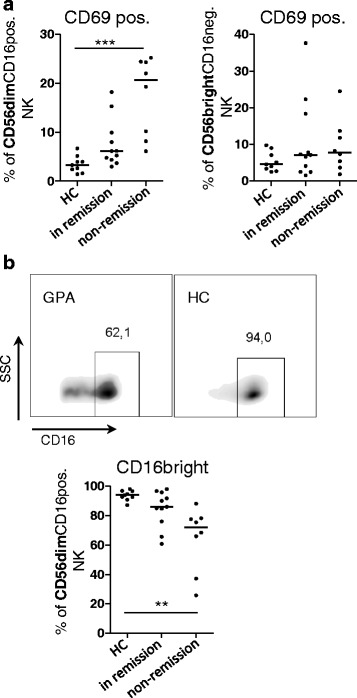
CD56^dim^ natural killer (NK) cells in active granulomatosis with polyangiitis (*GPA*) express high levels of lymphocyte activation marker CD69 and low levels of Fc-gamma receptor CD16. Experimental setting, patients and analysis as described in Fig. 3, using multicolor panel I. As this panel included an anti-CD56 antibody with low sensitivity for the discrimination between ^dim^ and ^bright^ but also anti-CD16, we redefined 'CD56^dim^' NK cells as 'CD56^dim^(CD16 pos.)' NK cells and 'CD56^bright^' NK cells as 'CD56^bright^(CD16 neg.)' NK cells. **a** *Left* percentages of CD69-positive CD56^dim^(CD16 pos.) NK cells; Kruskal-Wallis test, *p* = 0.0003. *Right* percentages of CD69-positive CD56^bright^(CD16 neg.) NK cells; Kruskal-Wallis test, not significant. **b** *Top* examples of *dot blots* show CD16 expression in healthy controls (*HC*) and patients with GPA and the definition of 'CD16^bright^ CD56^dim^(CD16 pos.) NK cells'. *Bottom* percentages of CD16^bright^ CD56^dim^(CD16 pos.); Kruskal-Wallis test, *p* = 0.0003. **a**, **b** Dunn's post hoc test was significant where indicated in the graphs. *SSc* side scatter

CD56^dim^(CD16 pos.) NK cells more frequently expressed CD69 in active GPA (Fig. 4a, left graph). CD69 expression was also slightly increased in remission (Dunn's post hoc test not significant; Mann-Whitney test, *p* = 0.0122). On the contrary, CD69 on CD56^bright^(CD16 neg.) NK cells was not different between HC and patients with GPA (Fig. 4a, right graph).

The activation of NK cells via CD16 leads to the downregulation of surface CD16, according to previous literature. Under healthy conditions, >90 % of CD56^dim^(CD16 pos.) NK cells express CD16 in a CD16^bright^ fashion (Fig. 4b). In active GPA, the proportions of CD16^bright^ CD56^dim^(CD16 pos.) NK cells were significantly lower (Fig. 4b). The means of CD16 fluorescence intensity were significantly lower in active GPA than in remission (*p* = 0.0044, not shown). Together, these data demonstrate that the numerically decreased CD56^dim^ NK cells are activated in active GPA. CD56^bright^ NK cells, which do not express CD16 [20], were not activated. The following analysis therefore concentrates on CD56^dim^ NK cells.

### The expression of numerous NK cell receptors was not different in HC and GPA in remission or active GPA

CD56^dim^ NK cells can be further subgrouped by the expression of the maturity markers CD62L and CD57. CD62L was slightly increased and CD57 was slightly decreased on CD56^dim^ NK cells in both GPA in remission and active GPA (not shown, not statistically significant). The expression of CD69 was not different between CD57 and CD62L-positive CD56^dim^(CD16 pos.) NK cells (not shown). NKp30, NKp44, NK46, NKG2D and DNAM1 did not differ between HC and patients with GPA in remission or non-remission (Additional file 3: Figure S1). In addition, 2B4, CRACC and 41BB were unaltered (not shown).

### NK cell phenotype in relation to immunosuppressive treatment

Prednisone dosage was not significantly different between patients in remission and non-remission (Additional file 4: Figure S2A). There was no correlation between prednisone dosage and the proportion of CD16^bright^ and CD69 pos. CD56^dim^(CD16 pos.) NK cells (Additional file 4: Figure S2B).

Apart from prednisone, patients with GPA took diverse immunosuppressive drugs (Additional file 4: Figure S2C). We compared the proportion of CD16^bright^ and CD69 pos. CD56^dim^ (CD16 pos.) NK cells after subgrouping according to immunosuppressive drugs (Additional file 4: Figure S2D and E, left graphs). On statistical analysis using the Kruskal-Wallis test, there was no statistical difference between the patient groups. The Kruskal-Wallis test was only positive after the inclusion of the HC group; the only positive post hoc tests were comparisons between patients who received azathioprine and HC. In the azathioprine group, 4/ 6 patients were not in remission. Comparing patients receiving azathioprine and rituximab according to their disease activity, we observed a clear tendency towards phenotypic alterations that were more prominent in patients with active GPA (Additional file 4: Figure S2D and E, right graph).

### Increased expression of adhesion molecule CD54 (ICAM-1), chemokine receptor CCR5 and NKG2C on CD56^dim^ NK cells in active GPA

**Fig. 5:**
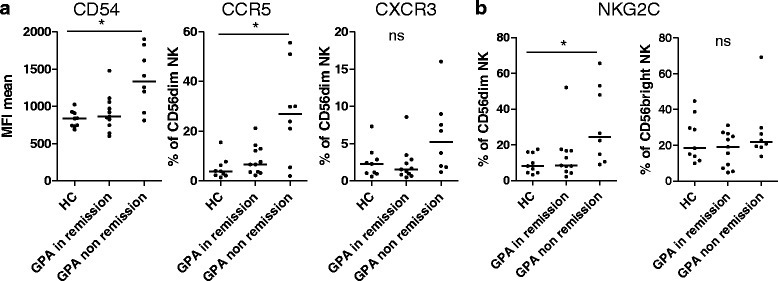
Increased expression of adhesion molecule CD54 (ICAM-1), chemokine receptor CCR5 and NKG2C on CD56^dim^ natural killer (*NK*) cells in active (non-remission) granulomatosis with polyangiitis (*GPA*). Experimental setting, patients and analysis as described in Fig. 3, using multicolor panel III. **a** *Left* mean fluorescent intensity (*MFI*) of CD54 on CD56^dim^ NK cells; Kruskal-Wallis test, *p* = 0.0108; *middle* percentage of CCR5-positive CD56^dim^ NK cells (Kruskal-Wallis test, *p* = 0.0204); *right* percentage of CXCR3-positive CD56^dim^ NK cells (Kruskal-Wallis test not significant). **b** *Left* percentage of NKG2C on CD56^dim^ NK cells (Kruskal-Wallis test, *p* = 0.0227); *right* percentage of NKG2C on CD56^bright^ NK cells (Kruskall-Wallis test not significant). **a**, **b** Dunn's post hoc test was significant where indicated in the graphs. *HC* healthy controls

CD54 was expressed on all CD56^dim^ NK cells, which did not differ between HC and patients with GPA (not shown). However, the mean fluorescent intensity of CD54 was significantly increased in active GPA (Fig. 5a).

CCR5 was not expressed, or was expressed at low levels on CD56^dim^ NK cells in HC, but the percentage of CCR5 pos. CD56^dim^ NK cells was significantly higher in patients with active GPA than in HC (Fig. 5a). CXCR3 tended to be increased in active GPA but was not statistically significant (Fig. 5a). NKG2C was expressed at low levels (0–20 %) on HC CD56^dim^ NK cells, but was significantly increased in active GPA (Fig. 5b). NKG2C expression on CD56^bright^ NK cells was not different between HC and patients with GPA.

Next we investigated whether CD54, CCR5 and NKG2C were co-expressed on CD56^dim^ NK cells. CD54 and CXCR3 were not higher on NKG2C-positive than on NKG2C-negative CD56^dim^ NK cells (Additional file 5: Figure S4). Despite being significantly more frequently expressed on NKG2C-positive cells, CCR5 was also found on NKG2C-negative cells (Additional file 5: Figure S4). Therefore, these receptors were not increased on the same cells. To summarize, the expression levels of CD54, CCR5 and NKG2C were independently increased on CD56^dim^ NK cells in active GPA.

### Natural cytotoxicity was significantly decreased in active GPA

**Fig. 6:**
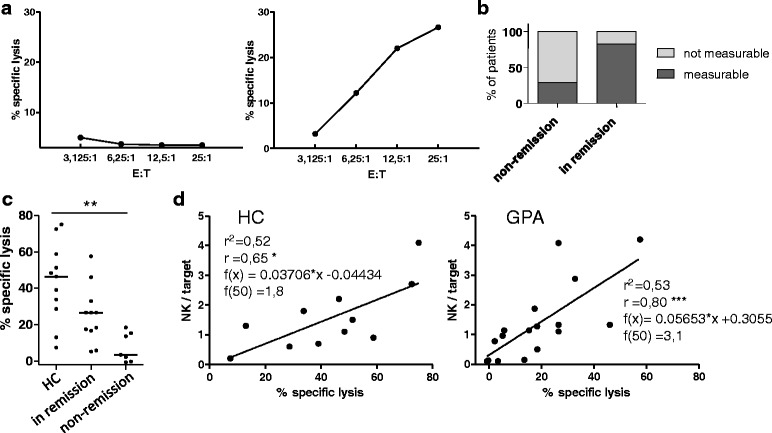
Natural cytotoxicity is not measurable in active (non-remission) granulomatosis with polyangiitis (*GPA*). Freshly isolated, thawed peripheral blood mononuclear cells (PBMCs) from 11 healthy controls (*HC*) and patients with GPA from cohort II (in remission = 11 and non-remission = 7 patients; total = 18) were cultured overnight in medium. After washing PBMCs (effector cells) and ^51^Cr-labeled K562 (target) cells were co-cultured for 4 hours without further stimulation. The highest effector (PBMC)/target (*E:T*) ratio was 25:1, only viable PBMCs were counted. Percent specific lysis was determined by measuring ^51^Cr in the supernatant. The sensitivity threshold of this assay lies at about 5–10 % specific lysis. **a** No natural cytotoxicity was detectable in PBMCs from 7/18 patients (*left*), whereas typical cytotoxicity curves were measured in PBMCs from the remaining 11/18 patients and all HC (*right*). **b** Analysis of the GPA activity states in patients revealed that PBMCs without natural cytotoxic response were all from patients in the non-remission group, except for the two patients with the lowest natural killer (*NK*)/target ratios within the remission group (0.96 and 1.1, respectively). Two patients from the non-remission group exhibited measurable minor natural cytotoxicity; both were clinically categorized as having "grumbling disease". **c** Statistical analysis at the highest E (viable PBMC):T ratio (25:1); Kruskal-Wallis test, *p* = 0.0016; Dunn's post hoc test was significant where indicated in the graph. **d** NK/target ratios of the highest E:T were calculated based on the percentages of NK cells in PBMCs and blotted against the percentage of lysed target cells (% specific lysis); linear equation, *r* ^2^, Spearman's *r* and statistical significance of the correlation as indicated in the graphs

PBMCs from 18 different patients with GPA in cohort II were thawed and co-cultured with ^51^Cr-labeled K562 target cells. The cytotoxic response after 4 hours of co-culture was not detectable in the majority of PBMCs from patients with active GPA (*n* = 7) (Fig. 6a, left graph), whereas the PBMCs from HC and from the majority of patients in remission (*n* = 11) responded typically to K562 targets (Fig. 6a, right graph). For further comments on single patients, see the legend of Fig. 6b. On statistical analysis of the highest effector (viable PBMC)/target ratio, PBMCs from active GPA had significantly reduced natural cytotoxicity (Fig. 6c). Differences in NK cell percentages within the PBMCs (shown in Fig. 2) could account for this effect. Additionally, NK cell activity per se could be altered in patients with active GPA. We therefore calculated the number of NK cells within the PBMCs for each sample. In both HC and patients with GPA, there was good correlation between the percentage of lysed target cells and the calculated NK/target ratio (Fig. 6d). A linear regression model of the relationship between target cell lysis and the NK/target ratio revealed that more GPA NK cells would be needed to obtain the same target cell lysis as HC NK cells (Fig. 6d): to reach a 50 % target cell lysis, an NK/target ratio of 1.8 was statistically sufficient in HC, but a ratio of 3.1 was needed in patients with GPA.

### NK cell cytotoxicity in relation to immunosuppressive treatment

There was no correlation between prednisone dosage and cytotoxicity (Additional file 4: Figure S2B). We compared the cytotoxicity after subgrouping according to immunosuppressive drugs (Additional file 4: Figure S2F). Similar to what was observed in the expression of CD16 and CD69 on CD56^dim^ (CD16 pos.) NK cells, on statistical analysis using the Kruskal-Wallis test there was no statistic difference between the patient groups. The Kruskal-Wallis test was only positive after the inclusion of the HC; the only positive post hoc test was again the comparison between HC and the six patients who received azathioprine (four of whom were not in remission). Comparing patients receiving azathioprine and rituximab according to GPA activity, we observed a clear tendency towards alterations in cytotoxicity that were more prominent in patients in the non-remission group (Additional file 4: Figure S2F).

In summary, the natural cytotoxicity of active PBMCs was reduced to incommensurability, which can be traced back to low NK cell numbers. In addition, we observed decreased cytotoxicity on the re-calculated per-cell level.

## Discussion

Despite their important function in immune defense and regulation, the role of NK cells in many autoimmune or inflammatory diseases remains unexplored. Only recently have the first publications on NK cells in GPA emerged [15, 21]. Importantly, in our previous study peripheral blood NK cell percentages in long-term remission were significantly higher than in active GPA and control groups [15]. These clinical correlations warranted further investigation.

In the first part of this study, we addressed this issue by adding a second cohort of patients, providing both absolute and relative lymphocyte subset counts. We confirmed normal NK cells percentages in active GPA and high NK cell percentages in long-term remission, despite increased patient numbers and slightly altered analysis methods. Next, we reported, for the first time, lower absolute NK cell counts in non-remission vs. remission GPA. It is important to note that while overall B cell and T cell lymphopenia persist during active and inactive GPA, the absolute NK cell counts were significantly higher in remission.

In addition, the majority of patients with non-remission GPA had NK cell counts lower than the clinically established normal threshold, indicating a true decrease in NK cells in non-remission GPA. This true decrease was not formally confirmed when comparing non-remission GPA with inactive CD, as the corresponding post hoc test was not significant. However, the direct comparison of non-remission GPA and CD using the Mann-Whitney test was significant. This borderline significance is presumably due to the relatively low number of patients with CD and the large range of scattered NK counts within the CD group.

The follow up during induction therapy suggests an intra-individual increase in NK cells over time. In line with this, we observed a tendency towards a decrease in NK cell percentages during flaring disease activity among patients who were investigated at different time points in both cohorts. Together, these data sustain the inverse correlation between NK cells and the disease course.

In the second part of this study, we systematically analyzed NK cell subsets and their phenotype. The CD56^dim^ subset was identified as the reduced and altered NK cell fraction in active GPA. There were no significant differences on further subtyping of CD56^dim^ NK cells according to the maturity markers CD62L and CD57. CD56^dim^ NK cells are characterized by the expression of the Fc-receptor CD16. Importantly, CD16^bright^ cells were strongly reduced in active GPA. This alteration is prominent, given the importance of the receptor [11]. In addition, CD16 polymorphisms have been identified as a risk factor in GPA [22]. CD56^dim^(CD16 pos.) NK cells also showed signs of acute (CD69 and CD54) and chronic (NKG2C) activation, and de novo expression of CCR5 in active GPA. The normal expression patterns of the majority of the investigated NK cell receptors (Additional file 3: Figure S1) confirms the specificity of these phenotypic changes. Upon cytomegalovirus (CMV) reactivation, NKG2C-positive NK cells increase; however, only two patients in our patient cohort had documented CMV reactivation within recent years.

Alternative to the disease and/or its activity, phenotypic changes may be due to medication. The dissection of treatment and/or disease-mediated alterations observed in ex vivo biomaterial is a known challenge (confounder by treatment vs. confounder by indication/disease activity). Based on our treatment analysis (Additional file 4: Figure S2), we conclude that a confounder by treatment in the sense of a general effect of immunosuppression cannot be completely excluded, but effects were not drug-specific. Disease activity seems to be a major confounder of the only statistically significant post hoc test that could be detected between treatment groups, i.e. patients taking azathioprine vs. HC. In addition to that, the tendency of more pronounced alterations in patients with active vs. inactive GPA who received the same drugs clearly indicates that disease activity is closely related to the phenotypic and functional alterations observed in this study.

Together, these findings suggest the involvement of NK cells in active GPA. In this context, two possible explanations for decreased NK cell counts could exist, namely different migration and activation-induced NK cell death:

1. The elevation of the adhesion molecule CD54 and the chemokine receptor CCR5 might be indicative of a difference in migration. It is unclear, into which tissues NK cells would migrate, as we did not detect NK cells in granulomas [15]. Alternatively, NK cells could die in granulomas, or migrate into secondary lymphoid tissues
2. The activated phenotype and the downregulation of CD16 could be a sign of Fc-induced activation of NK cells (Merkt et al. 2016, unpublished observations) [14], e.g. by cell-bound autoantibodies. Alternatively, CD16 downregulation could be secondary to induced matrix metalloproteinases [12, 13]. GPA is a classic autoimmune disease characterized by the existence of autoantibodies, and chronic stimulation by cell-bound autoantibodies could lead to activation-induced cell death and thus, reduced NK cell numbers

These considerations could be combined into an integrative model: CD16 of peripheral blood CD56^dim^ NK cells could recognize proteinase3-autoantibodies bound on endothelial cells, which activates them and increases the expression of CD54 (and presumably CCR5). As a result, they might transmigrate through the blood vessel wall, sustaining endothelial damage, and die due to activation-induced cell death. After induction therapy, autoantibodies and activating chemokines may decrease [23], resulting in reduced NK cell migration and death, leading to increased counts in peripheral blood.

This model gives no explanation why NK cell percentages further increase during long-term remission. Increased NK cell proportions might include changes in NK cell maturation, development, proliferation or survival. Based on the existing literature, elevated blood levels of IL-15 and IL-18 [23], a disturbed control by T regulatory cells [24] and different migration due to changes of chemotactic receptors (Fig. 5 and [25]) also need to be considered. Based on mouse models, it had been suggested that lymphopenia itself could also be a stimulus for NK cell proliferation [8]. Elevated proportions in remission and decreased counts in active GPA are likely to be the result of two distinct mechanisms, because phenotypic alterations were more prominent in active GPA. Accordingly, NK cells might have different roles in remission and active GPA.

In the last part of this study, we described overall deficiency of natural cytotoxicity towards the classic NK cell target K562 in active GPA. The killing of K562 is independent of CD16 and depends on NKG2D, the ligand of which has been detected in the tissues in GPA [26, 27], and on inflammatory endothelial cells [28]. Therefore, the reduced natural cytotoxicity towards K562 might be relevant in GPA. Based on the correlation between lysed K562 cells and NK cell counts, the reduced NK cell numbers in active GPA are responsible for the dysfunctional overall natural killing. However, we also observed a reduced killing capacity at the re-calculated per-cell level in GPA in general, indicating that a second mechanism could lead to reduced natural cytotoxicity. However, the latter finding needs validation using isolated NK cells, which could not be done in the present study because of a limited amount of available PBMCs.

While several studies [29] report decreased NK cell numbers and impaired cytotoxicity in other autoimmune diseases, there is a lack of studies in GPA. Systemic lupus erythematosus (SLE) is such another disease. Hervier et al. [30] describe numerical, phenotypic and functional alterations in NK cells in SLE that are at least in part similar to those reported here in GPA, including downregulated CD16 and upregulated CD69 and NKG2C. Therefore, some of our findings could be a general feature of active autoantibody-mediated systemic inflammatory diseases. These similarities are encouraging, as they might reflect (measurable) disease activity, and comparisons might open new strategies for common or specific treatment targets. In any case, increased NK cell percentages in remission above HC levels have, to the best of our knowledge, not been described in other diseases and appear to be characteristic of GPA [15].

Together, the alterations in active GPA implicate a role in GPA pathogenesis. In the long run, NK cell numbers and their phenotype (CD16, CD69, NKG2C) or natural cytotoxicity are promising candidates to serve as clinical biomarkers to determine GPA activity. In addition, the described phenotypic changes might help to form a basis for new therapeutic strategies in GPA.

## Conclusions

In summary, we provided further evidence of correlation between blood NK cells and clinical suppression of disease activity. We described for the first time that reduced numbers of CD56^dim^ NK cells in active GPA exhibit an activated and altered phenotype and are associated with deficient overall natural cytotoxicity.

## Additional files

Additional file 1: Antibody/staining panels. (DOCX 13 kb) Additional file 2: Figure S3. Age was not different between HC, CD and patients with active or inactive (non-remission) GPA in NK cell phenotype analysis. Shown are patients and HC as presented in Figs. 3, 4 and 5 and patients with CD as shown in Fig. 2. *Bars* indicate medians. The Kruskal-Wallis test revealed no significant differences between the groups. *Y-axes* show age in years. (PDF 5 kb) Additional file 3: Figure S1. The expression of numerous NK cell receptors is not different in HC and patients with GPA in remission or with active GPA. The percentage of the indicated receptors on CD56^dim^ NK cells is shown for healthy controls (*HC*) and GPA in remission (*ir*) and non-remission (*nr*). Statistical analysis using the Kruskal-Wallis test revealed no significant differences. The percentage of NKG2D-positive NK cells is underestimated due to weak fluorescence intensity of the FITC-labeled antibody; the example of a histogram shows that almost all CD56^dim^ NK cells express NKG2D. (PDF 112 kb) Additional file 4: Figure S2 NK cell phenotype and cytotoxicity in relation to immunosuppressive treatment. Patients and methods as indicated in Fig. 4. a, b Prednisone dosages. a Prednisone dosages were not significantly (*ns*) different between patients in remission and in non-remission. b There was no correlation between prednisone dosage and the proportion of CD16^bright^ and CD69 pos. CD56^dim^CD16 pos. NK cells (for experimental details see Fig. 3) or cytotoxicity (% specific lysis, for experimental details see Fig. 6). c-f Other immunosuppressive drugs. c *CYC* cyclophosphamide, *RTX* rituximab (rituximab was given 7.3 (remission) and 8.3 (non-remission) months (means) prior to inclusion in the study), *AZA* azathioprine, *MTX* methotrexate, *LEF* leflunomide (AZA, MTX and LEF were introduced >/= 5 months prior to the inclusion in the study; the starting point of LEF from a could not be retrieved for one patient), *double IS* combination therapies, *no IS* no immunosuppressive drugs apart from prednisone. Of note, AZA was more often taken by patients in non-remission, whereas MTX, LEF and MPA were more often taken by patients in remission. d-f *Left* graphs show the frequencies of CD16^bright^ CD56^dim^CD16 pos. NK cells, CD69 pos. CD56^dim^CD16 pos. NK cells and cytotoxicity (% specific lysis) after subgrouping according to immunosuppressive drugs apart from prednisone, respectively. Statistical analysis using the Kruskal-Wallis test revealed that there was no statistical difference between patient groups. Upon inclusion of HC, the Kruskal-Wallis test was positive; the only positive post hoc tests were HC vs. patients who received azathioprine. For two treatment groups (rituximab and azathioprine) patients in remission (*black dots*) were opposed to those in non-remission (*red dots*). The remaining treatment groups were not depicted as only each one patient or no patient at all were in non-remission. *Bars* represent medians. (PDF 109 kb) Additional file 5: Figure S4. CD54, CCR5 and NKG2C were not increased on the same CD56^dim^ NK cells. Experimental setting, patients and analysis as described in Fig. 5. The mean MFI of CD54 on CD56^dim^ NK cells and the percentages of CCR5-positive and CXCR3-positive CD56^dim^ NK cells depending on the co-expression of NKG2C are shown. The same donors are linked by *lines*. The Wilcoxon signed rank test was significant where indicated in the graph; **p* = 0.0156. (PDF 169 kb)

## Abbreviations

2B4: CD244
41BB: CD137
51 Cr: 51-Chrome
BVAS: Birmingham vasculitis activity score
CCR5: C-C motif receptor type 5
CD(with number): Cluster of differentiation
CD: control disease
CD16: Fc-gamma receptor IIIA
CRACC: CD319
CXCR3: C-X-C motif receptor type 3
DMARD: disease modifying antirheumatic drug
DNAM1: CD226, DNAX accessory molecule-1
Fc: fragment c
FCS: fetal calf serum
GPA: granulomatosis with polyangiitis
HC: healthy control
ICAM1: CD54
IL: interleukin
NK cell: natural killer-cell
NKG2C: natural-killer group 2, member D activating NK-cell receptor
NKG2D: natural-killer group 2, member D activating NK-cell receptor
NKp30/NKp44/NKp46: natural killer - cell p30/44/46-related protein
ns: not significant
PBL: peripheral blood lymphocyte
PBMC: peripheral blood mononuclear cell
PBS: phosphate-buffered saline
SLE: systemic lupus erythematosus

## References

[1] Vivier E (2011). Innate or adaptive immunity? The example of natural killer cells. Science.

[2] Watzl C (2014). Natural killer cell regulation - beyond the receptors. F1000Prime Rep.

[3] Vivier E (2008). Functions of natural killer cells. Nat Immunol.

[4] Colonna M, Jonjic S, Watzl C (2011). Natural killer cells: fighting viruses and much more. Nat Immunol.

[5] Tian Z, Gershwin ME, Zhang C (2012). Regulatory NK cells in autoimmune disease. J Autoimmun.

[6] Vivier E, Ugolini S (2009). Regulatory natural killer cells: new players in the IL-10 anti-inflammatory response. Cell Host Microbe.

[7] Cerboni C (2007). Antigen-activated human T lymphocytes express cell-surface NKG2D ligands via an ATM/ATR-dependent mechanism and become susceptible to autologous NK- cell lysis. Blood.

[8] Sun JC, Lanier LL (2011). NK cell development, homeostasis and function: parallels with CD8(+) T cells. Nat Rev Immunol.

[9] Watzl C, Long EO. Signal transduction during activation and inhibition of natural killer cells. Curr Protoc Immunol. 2010;Chapter 11:p. Unit 11 9B. 10.1002/0471142735.im1109bs90 PMC3857016 20814939

[10] Nimmerjahn F, Ravetch JV (2008). Fcgamma receptors as regulators of immune responses. Nat Rev Immunol.

[11] Bryceson YT (2006). Synergy among receptors on resting NK cells for the activation of natural cytotoxicity and cytokine secretion. Blood.

[12] Grzywacz B, Kataria N, Verneris MR (2007). CD56(dim)CD16(+) NK cells downregulate CD16 following target cell induced activation of matrix metalloproteinases. Leukemia.

[13] Peruzzi G (2013). Membrane-type 6 matrix metalloproteinase regulates the activation-induced downmodulation of CD16 in human primary NK cells. J Immunol.

[14] Capuano C (2015). Anti-CD20 therapy acts via FcgammaRIIIA to diminish responsiveness of human natural killer cells. Cancer Res.

[15] Merkt W (2015). Peripheral blood natural killer cell percentages in granulomatosis with polyangiitis correlate with disease inactivity and stage. Arthritis Res Ther.

[16] Hellmich B (2015). Treatment strategies for ANCA-associated vasculitides. Z Rheumatol.

[17] Hellmich B (2007). EULAR recommendations for conducting clinical studies and/or clinical trials in systemic vasculitis: focus on anti-neutrophil cytoplasm antibody-associated vasculitis. Ann Rheum Dis.

[18] Mukhtyar C (2009). EULAR recommendations for the management of primary small and medium vessel vasculitis. Ann Rheum Dis.

[19] Ntatsaki E (2014). BSR and BHPR guideline for the management of adults with ANCA-associated vasculitis. Rheumatology (Oxford).

[20] Cooper MA, Fehniger TA, Caligiuri MA (2001). The biology of human natural killer-cell subsets. Trends Immunol.

[21] Tognarelli S (2014). Tissue-specific microvascular endothelial cells show distinct capacity to activate NK cells: implications for the pathophysiology of granulomatosis with polyangiitis. J Immunol.

[22] Dijstelbloem HM (1999). Fcgamma receptor polymorphisms in Wegener's granulomatosis: risk factors for disease relapse. Arthritis Rheum.

[23] Monach PA (2013). Serum proteins reflecting inflammation, injury and repair as biomarkers of disease activity in ANCA-associated vasculitis. Ann Rheum Dis.

[24] Abdulahad WH (2007). Functional defect of circulating regulatory CD4+ T cells in patients with Wegener's granulomatosis in remission. Arthritis Rheum.

[25] Kottilil S (2004). Expression of chemokine and inhibitory receptors on natural killer cells: effect of immune activation and HIV viremia. J Infect Dis.

[26] de Menthon M (2011). Excessive interleukin-15 transpresentation endows NKG2D + CD4+ T cells with innate-like capacity to lyse vascular endothelium in granulomatosis with polyangiitis (Wegener's). Arthritis Rheum.

[27] Capraru D (2008). Expansion of circulating NKG2D+ effector memory T-cells and expression of NKG2D-ligand MIC in granulomaous lesions in Wegener's granulomatosis. Clin Immunol.

[28] Holmen C (2005). Circulating inflammatory endothelial cells contribute to endothelial progenitor cell dysfunction in patients with vasculitis and kidney involvement. J Am Soc Nephrol.

[29] Fogel LA, Yokoyama WM, French AR (2013). Natural killer cells in human autoimmune disorders. Arthritis Res Ther.

[30] Hervier B (2011). Phenotype and function of natural killer cells in systemic lupus erythematosus: excess interferon-gamma production in patients with active disease. Arthritis Rheum.

