# Supplementary material for: Active but not inactive granulomatosis with polyangiitis is associated with decreased and phenotypically and functionally altered CD56dim natural killer cells
Source: Arthritis Res Ther. 2016 Sep 13;18(1):204. doi: 10.1186/s13075-016-1098-7 (PMC5022237; doi:10.1186/s13075-016-1098-7)
Supplement: Additional file 1: — Antibody/staining panels. (DOCX 13 kb) [file 13075_2016_1098_MOESM1_ESM.docx]

Additional file 1: Methods:

All antibodies were purchased from BD Biosciences, San Jose, CA, USA, if not indicated otherwise. The following antibody/staining panels were used: *Panel I*: anti-CD62L Brilliant Violet 421, anti-CD16 FITC, anti-CD3 PerCP (Biolegend, San Diego, CA, USA), anti-CD57 PE, anti-CD56 APC, anti-CD69 APC-H7. *Panel II*: anti-CD56 Brilliant Violet 421, anti-DNAM1 FITC, anti-CD3 PerCP (Biolegend, San Diego, CA, USA), anti-NKp44 PE, anti-NKp46 PE/Cy7, anti-NKp30 Alexa Fluor 647, anti-NKG2D Alexa Fluor 700 (R&D Systems, Minneapolis, MN, USA). *Panel III*: anti-CD56 Brilliant Violet 421, anti-NKG2C Alexa Fluor 488 (R&D Systems, Minneapolis, MN, USA), anti-CD3 PerCP (Biolegend, San Diego, CA, USA), anti-CD137 (41BB) PE (Biolegend, San Diego, CA, USA), anti-CXCR3 PE/Cy7 (Biolegend, San Diego, CA, USA), anti-CD54 Alexa Fluor 647 (Biolegend, San Diego, CA, USA), anti-CCR5 APC-Cy7 (Biolegend, San Diego, CA, USA). *Panel IV*: anti-CD56 Brilliant Violet 421, anti-CD244(2B4) FITC (Biolegend, San Diego, CA, USA), anti-CD28 PerCP/Cy5.5, anti-CD319 (CRACC, CS1) PE (Biolegend, San Diego, CA, USA), anti-CD3 Alexa Fluor 647 (Biolegend, San Diego, CA, USA), anti-CD4 APC-H7.
