# Supplementary figures and images for: Active but not inactive granulomatosis with polyangiitis is associated with decreased and phenotypically and functionally altered CD56dim natural killer cells
Source: Arthritis Res Ther. 2016 Sep 13;18(1):204. doi: 10.1186/s13075-016-1098-7 (PMC5022237; doi:10.1186/s13075-016-1098-7)

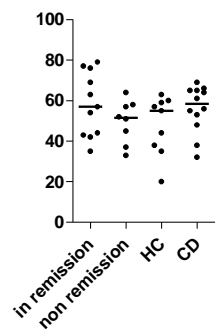

Supplement: Additional file 2: Figure S3. — Age was not different between HC, CD and patients with active or inactive (non-remission) GPA in NK cell phenotype analysis. Shown are patients and HC as presented in Figs. 3, 4 and 5 and patients with CD as shown in Fig. 2. Bars indicate medians. The Kruskal-Wallis test revealed no significant differences between the groups. Y-axes show age in years. (PDF 5 kb) [file 13075_2016_1098_MOESM2_ESM.pdf]

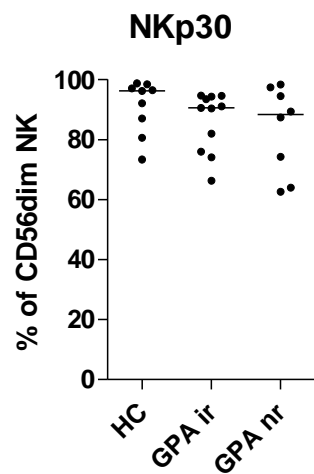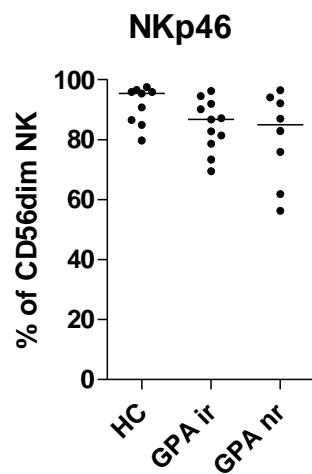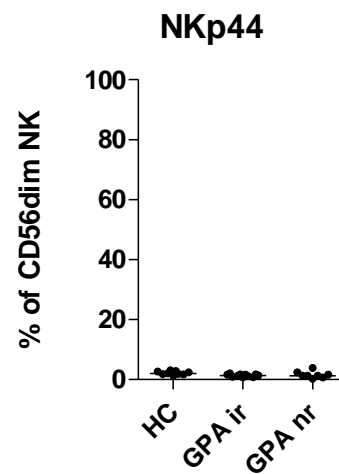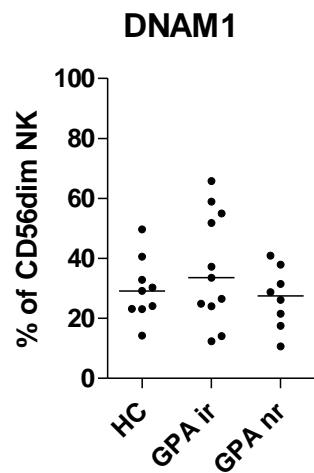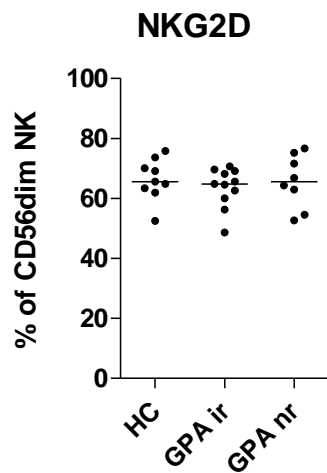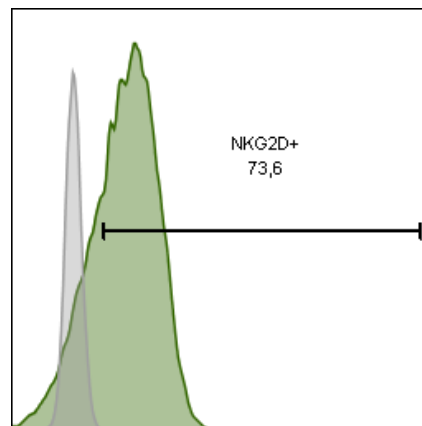

Supplement: Additional file 3: Figure S1. — The expression of numerous NK cell receptors is not different in HC and patients with GPA in remission or with active GPA. The percentage of the indicated receptors on CD56dim NK cells is shown for healthy controls (HC) and GPA in remission (ir) and non-remission (nr). Statistical analysis using the Kruskal-Wallis test revealed no significant differences. The percentage of NKG2D-positive NK cells is underestimated due to weak fluorescence intensity of the FITC-labeled antibody; the example of a histogram shows that almost all CD56dim NK cells express NKG2D. (PDF 112 kb) [file 13075_2016_1098_MOESM3_ESM.pdf]

A

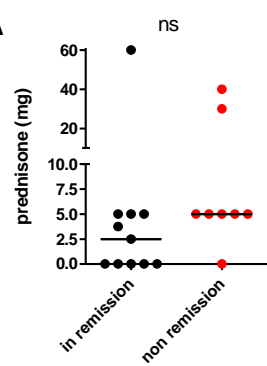

B

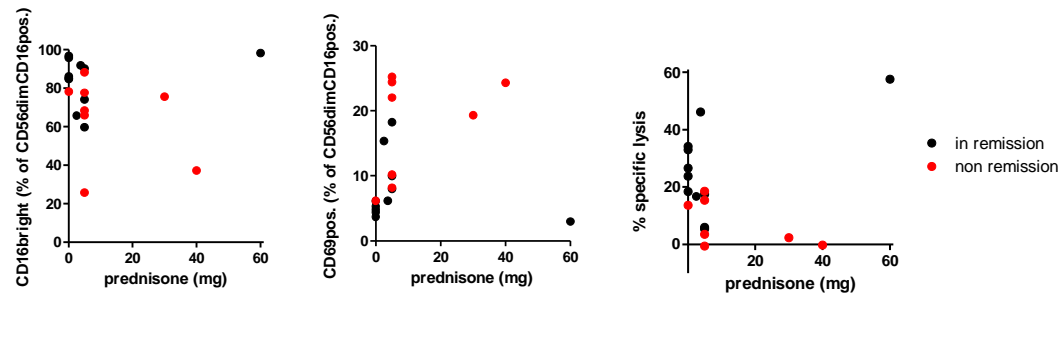

C

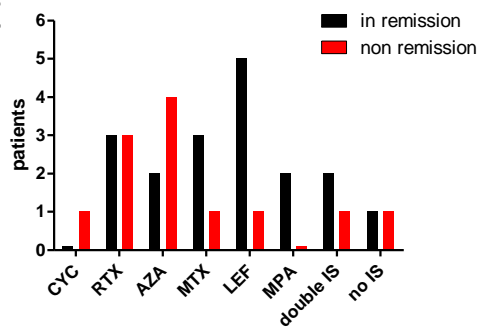

D

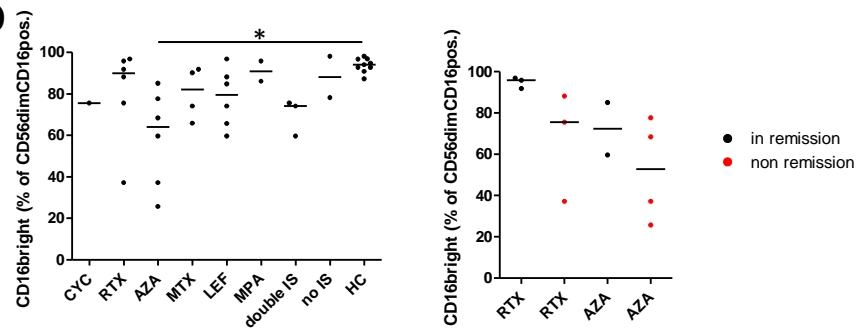

E

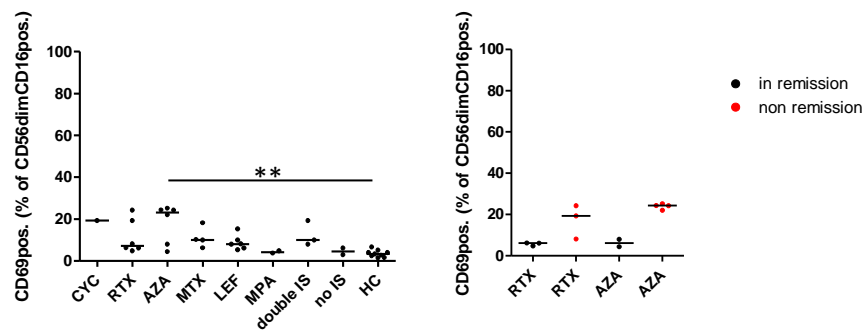

F

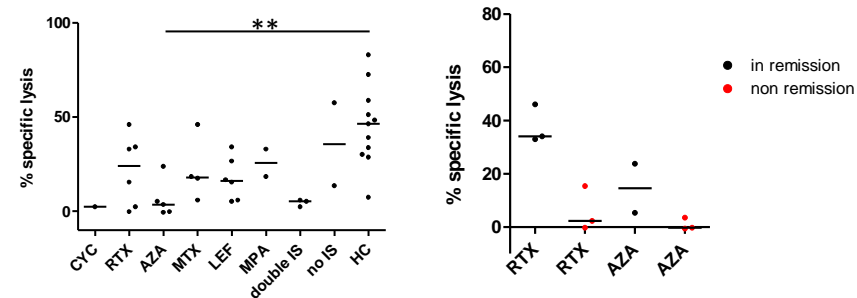

Supplement: Additional file 4: Figure S2 — NK cell phenotype and cytotoxicity in relation to immunosuppressive treatment. Patients and methods as indicated in Fig. 4. a, b Prednisone dosages. a Prednisone dosages were not significantly (ns) different between patients in remission and in non-remission. b There was no correlation between prednisone dosage and the proportion of CD16bright and CD69 pos. CD56dimCD16 pos. NK cells (for experimental details see Fig. 3) or cytotoxicity (% specific lysis, for experimental details see Fig. 6). c-f Other immunosuppressive drugs. c CYC cyclophosphamide, RTX rituximab (rituximab was given 7.3 (remission) and 8.3 (non-remission) months (means) prior to inclusion in the study), AZA azathioprine, MTX methotrexate, LEF leflunomide (AZA, MTX and LEF were introduced >/= 5 months prior to the inclusion in the study; the starting point of LEF from a could not be retrieved for one patient), double IS combination therapies, no IS no immunosuppressive drugs apart from prednisone. Of note, AZA was more often taken by patients in non-remission, whereas MTX, LEF and MPA were more often taken by patients in remission. d-f Left graphs show the frequencies of CD16bright CD56dimCD16 pos. NK cells, CD69 pos. CD56dimCD16 pos. NK cells and cytotoxicity (% specific lysis) after subgrouping according to immunosuppressive drugs apart from prednisone, respectively. Statistical analysis using the Kruskal-Wallis test revealed that there was no statistical difference between patient groups. Upon inclusion of HC, the Kruskal-Wallis test was positive; the only positive post hoc tests were HC vs. patients who received azathioprine. For two treatment groups (rituximab and azathioprine) patients in remission (black dots) were opposed to those in non-remission (red dots). The remaining treatment groups were not depicted as only each one patient or no patient at all were in non-remission. Bars represent medians. (PDF 109 kb) [file 13075_2016_1098_MOESM4_ESM.pdf]

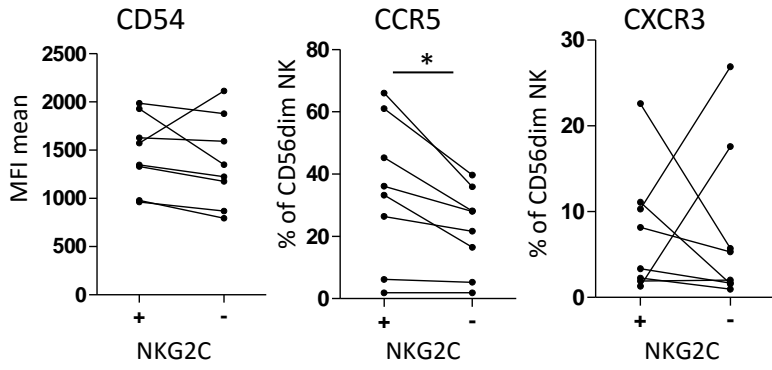

Supplement: Additional file 5: Figure S4. — CD54, CCR5 and NKG2C were not increased on the same CD56dim NK cells. Experimental setting, patients and analysis as described in Fig. 5. The mean MFI of CD54 on CD56dim NK cells and the percentages of CCR5-positive and CXCR3-positive CD56dim NK cells depending on the co-expression of NKG2C are shown. The same donors are linked by lines. The Wilcoxon signed rank test was significant where indicated in the graph; *p = 0.0156. (PDF 169 kb) [file 13075_2016_1098_MOESM5_ESM.pdf]
